# Additive Value of Biomarkers and Echocardiography to Stratify the Risk of Death in Heart Failure Patients with Reduced Ejection Fraction

**DOI:** 10.1155/2019/1824816

**Published:** 2019-05-02

**Authors:** Calogero Falletta, Francesco Clemenza, Catherine Klersy, Valentina Agnese, Diego Bellavia, Gabriele Di Gesaro, Chiara Minà, Giuseppe Romano, Pier Luigi Temporelli, Frank Lloyd Dini, Andrea Rossi, Claudia Raineri, Annalisa Turco, Egidio Traversi, Stefano Ghio

**Affiliations:** ^1^Cardiology Unit, Department for the Treatment and Study of Cardiothoracic Diseases and Cardiothoracic Transplantation, IRCCS-ISMETT (Istituto Mediterraneo per i Trapianti e Terapie ad alta specializzazione), Palermo, Italy; ^2^Service of Clinical Epidemiology & Biometry, Fondazione IRCCS Policlinico San Matteo, Pavia, Italy; ^3^Division of Cardiology, Istituti Clinici Scientifici Maugeri, IRCCS, Veruno, Italy; ^4^Cardiac, Thoracic and Vascular Department, University of Pisa, Pisa, Italy; ^5^Section of Cardiology, Department of Medicine, University of Verona, Verona, Italy; ^6^Division of Cardiology, Fondazione IRCCS Policlinico San Matteo, Pavia, Italy; ^7^Division of Cardiology, Istituti Clinici Scientifici Maugeri, IRCCS, Montescano, Italy

## Abstract

**Background:**

Risk stratification is a crucial issue in heart failure. Clinicians seek useful tools to tailor therapies according to patient risk.

**Methods:**

A prospective, observational, multicenter study on stable chronic heart failure outpatients with reduced left ventricular ejection fraction (HFrEF). Baseline demographics, blood, natriuretic peptides (NPs), high-sensitivity troponin I (hsTnI), and echocardiographic data, including the ratio between tricuspid annular plane excursion and systolic pulmonary artery pressure (TAPSE/PASP), were collected. Association with death for any cause was analyzed.

**Results:**

Four hundred thirty-one (431) consecutive patients were enrolled in the study. Fifty deaths occurred over a median follow-up of 32 months. On the multivariable Cox model analysis, TAPSE/PASP ratio, number of biomarkers above the threshold values, and gender were independent predictors of death. Both the TAPSE/PASP ratio ≥0.36 and TAPSE/PASP unavailable groups had a three-fold decrease in risk of death in comparison to the TAPSE/PASP ratio <0.36 group. The risk of death increased linearly by 1.6 for each additional positive biomarker and by almost two for women compared with men.

**Conclusions:**

In a HFrEF outpatient cohort, the evaluation of plasma levels of both NPs and hsTnI can contribute significantly to identifying patients who have a worse prognosis, in addition to the echocardiographic assessment of right ventricular-arterial coupling.

## 1. Introduction

Heart failure (HF) is the first cause of hospitalization and mortality in Western countries [1–3]. Clinical, laboratory, and instrumental parameters are currently applied in clinical practice to stratify prognosis of HF patients, either independently or aggregated in scores [4–7]. Clinicians require simple indicators to quickly stratify patient risk and manage therapeutic decisions, especially in outpatients. To this aim, echocardiography is probably the most useful resource, due to its wide availability, relatively low cost, and amount of information it provides [8–14]. The ratio between tricuspid annular plane systolic excursion and systolic pulmonary artery pressure (TAPSE/PASP) allows the noninvasive evaluation of systolic right ventricular (RV) function and pulmonary artery pressure. Accordingly, such a ratio provides a noninvasive estimation of the right ventricular- (RV-) arterial coupling [15]. A TAPSE/PASP ratio of <0.36 mm/mmHg is an independent predictor of mortality in HF patients with either reduced (HFrEF) or preserved (HFpEF) left ventricular ejection fraction [16]. Also, it has been shown that high plasma levels in biomarkers such as natriuretic peptides (NPs) and high-sensitivity troponin I (hsTnI) is associated with worse outcome in HF patients [17–20].

However, after the prognosis of chronic HF patients has been stratified with the echocardiographic evaluation of RV-arterial coupling based on TAPSE/PAPS ratio, it is unclear whether circulating biomarkers are still clinically useful at this aim. To verify this hypothesis is the goal of the present study.

## 2. Materials and Methods

### 2.1. Study Patients

This is a prospective, observational, multicenter study. Between November 2011 and September 2014, we prospectively enrolled a cohort of 431 consecutive clinically stable outpatients with chronic HF. Patients were recruited from three centers during routine clinical follow-up visits at the Heart Failure and Heart Transplant Units of the Cardiology Divisions of IRCCS San Matteo Hospital in Pavia, IRCCS-ISMETT in Palermo, and Istituti Clinici Scientifici Maugeri in Montescano. Inclusion criteria were HF outpatients with left ventricular ejection fraction (LVEF) ≤40% (HFrEF), in clinically stable conditions since at least 3 months. The exclusion criteria were congenital heart disease, severe organic valvular heart disease, a recent hospitalization for HF (<1 month), a recent myocardial revascularization or cardiac resynchronization therapy (CRT), device implant (<6 months), and severe renal failure requiring dialysis. Through interviews and examination of medical records performed at the moment of the enrollment, we collected the patients' demographic and clinical data as well as information on their medication. Similarly, we measured the patients' height, weight, and blood pressure. Blood samples were taken for measurement of plasma biomarkers. Also, a surface 12-lead electrocardiogram (EKG) was recorded. All patients gave informed written consent, and the Ethics Committee of each institution approved this study, which was carried out in compliance with the principles of the Declaration of Helsinki.

### 2.2. Biomarkers

Venous blood samples for NPs (brain natriuretic peptide (BNP) at the Pavia Center and/or N-terminal-proBNP (NT-proBNP) at the Palermo and Montescano centers) were drawn on the day of echocardiography. Chilled ethylenediaminetetraacetic acid (EDTA) tubes were centrifuged immediately at 4000 g (48°C) for 15 minutes. Separated plasma samples were processed by immunofluorescence assay (ADVIA Centaur platform, Siemens Healthcare Diagnostics S.R.L., Milan, Italy, for BNP; ECLIA; Roche Diagnostics; Indianapolis, Indiana, for NT-proBNP). For BNP, the lower assay detection limit was 1 pg/mL, while for NT-proBNP, the measuring range was 5–35000 pg/mL. We measured serum troponin I using a high-sensitive troponin I assay (Centaur TnI-Ultra/Siemens Medical Solution Diagnostics, NY). In this assay, the lower limit of detection is 0.006 ng/mL and the lowest concentration at which the coefficient of variation was <10% was 0.03 ng/ml, while the intra-assay and interassay CVs were 3.5% (*n*=4) and 4.2% (*n*=4), respectively, in a sample with a troponin I concentration of 0.03 ng/mL.

### 2.3. Echocardiography

Echocardiographic examinations were performed with Vivid System Seven (GE/Vingmed, Milwaukee, USA) at a 3.5 MHz ultrasound probe. All the exams were performed according to the most recent European recommendations [21]. Briefly, patients with an E-wave deceleration time (EDT) of 140 ms or less were classified as having a restrictive filling pattern [12]. Mitral regurgitation (MR) severity was quantified using vena contracta width, while tricuspid regurgitation (TR) was graded semiquantitatively (absent, trivial, mild, moderate, or severe) based on color flow imaging. Pulmonary artery systolic pressure (PASP) was obtained as the sum of pressure gradient between right atrium and right ventricle obtained from TR jet peak velocity, plus right atrial pressure (RAP) estimation combining inferior vena cava size and collapsibility. Tricuspid annular plane systolic excursion (TAPSE) was interpreted based on the most recent recommendations [21]. In patients who had both TAPSE and PASP values available, the TAPSE/PASP ratio was measured; if PASP estimation was not feasible due to the lack of TR jet, the ratio was not calculated. Accordingly, these patients were grouped separately (TAPSE/PASP unavailable).

### 2.4. Follow-Up and End Point of the Study

Patients underwent routine outpatient clinic visit every six months or earlier if needed. Patients who did not show up at scheduled visits were reached by phone. The primary end point of the study was death from any cause. Deaths were verified through hospital records and interviews with relatives.

### 2.5. Statistical Analysis

For the purpose of the analysis, we categorized the TAPSE/PASP ratio into 3 groups, according to the published cutoffs [14]: <0.36, ≥0.36, and unavailable. This value is confirmed as of the same order of the optimal cutoff at 24 months (value 0.37), identified with a time-dependent ROC curve analysis. Clinical, laboratory, and echocardiography data were summarized as mean and standard deviation (SD) or counts and percentage. The Kruskal–Wallis test or Fisher's exact test was used to compare groups, as appropriate. We computed the median follow-up (25th–75th percentiles) with the inverse Kaplan–Meier method. We computed mortality rates per 100 persons per year, with their 95% confidence intervals (95% CI). We plotted cumulative survival according to the Kaplan–Meier method. To provide clinicians with data that can be used in routine practice, we considered three main candidate predictors of prognosis: TAPSE/PASP ratio, NPs, and hsTnI plasma levels, together with EDT, age, and gender. These were chosen *a priori*, based on clinical expertise. Then, for the same purpose of simplicity and practical applicability, we dichotomized continuous predictors based on previously established prognostic meaning thresholds for patients with chronic HF [12, 14, 16, 22]. In particular, the threshold values were 0.027 pg/ml for hsTnI, 125 pg/ml for BNP, and 1016 pg/ml for NT-proBNP. A value of <140 msec for EDT and <0.36 for TAPSE/PASP ratio was chosen based on the literature [12, 16]. Age was dichotomized at its median of 66 years. The methods applied for the acquisition of information on all variables were exactly the same across the three clinical sites, except for the NPs used, as specified in Section 2.2. For this reason, in order to homogenize the data collected, we further collapsed BNP and NT-proBNP together as positive/negative NPs, based on their respective threshold. Finally, we generated the number of positive biomarkers (0, 1, or 2) among hsTnI and NPs. We fitted Cox regression models to assess the association of each predictor with survival. We computed the hazard ratio (HR) and 95% CI. We also planned subgroup analysis of the number of positive biomarkers within TAPSE/PASP ratio categories. Then, we included all candidate predictors in a multivariable Cox model to assess their independent prognostic values. We also fitted a second model without biomarkers for comparison. We computed the Harrell's *C* statistic to measure model discrimination (the closer to 1, the better) and derived 95% CI by bootstrapping. We also computed the Akaike information criterion for both models (the lower, the better). We tested the proportional hazard assumption based on Schoenfeld residuals; it was satisfied in all cases. A 2-sided *p* value was considered statistically significant. We used Stata 15 (StataCorp, College Station, TX, USA) for computation.

## 3. Results

### 3.1. Clinical and Echocardiographic Characteristics

**Figure 1:**
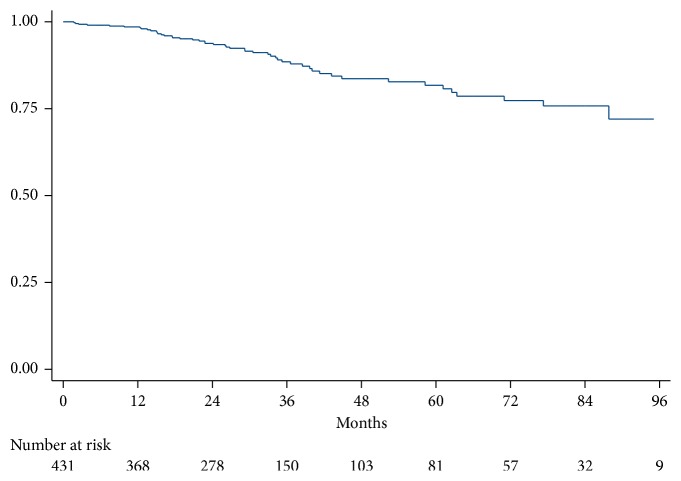
Kaplan–Meier curve illustrating the cumulative survival of the studied cohort. Numbers at risk are reported below the figure.

In the period of observation, 431 patients (83% males) met the inclusion criteria and were enrolled in the study (213, 196, and 22 patients for Pavia, Palermo, and Montescano, respectively). Mean age was 59 ± 12 years. At enrollment, patients were on optimized HF therapy. Overall population characteristics are summarized in Table 1. All the patients enrolled completed the follow-up until the end of the study or reaching the end point.  Figure 1 depicts cumulative survival of the entire study population. Patients are grouped in Table 2 according to the TAPSE/PASP ratio (<0.36, ≥0.36, or unavailable). The three groups did not differ in age and rate of comorbidities except for hypertension. However, patients with a TAPSE/PASP ratio of <0.36 displayed a higher prevalence of markers of advanced heart failure (an higher New York Heart Association (NYHA) class, a lower systolic blood pressure and level of hemoglobin; a lower EF, larger left atrial volume, a higher rate of restrictive EDT and presence of MR, a higher percentage of NPs values over the established threshold, and higher proportion of patients with both biomarkers above prognostic thresholds).

### 3.2. Predictors of Survival

Fifty deaths occurred over a median follow-up of 32 months (25th–75th: 20–53), corresponding to a mortality rate of 3.9 deaths per 100 persons per year (95% CI 2.9–5.1). Time to death was 26 months (25th–75th: 15–40). 11, 26, and 13 patients died in the TAPSE/PASP ratio <0.36, ≥0.36, and unavailable groups, respectively. Mortality rates in the TAPSE/PASP ratio ≥0.36 and unavailable groups were about 60% lower than those in the <0.36 group (Table 3, Cox model *p* < 0.001). Twelve patients were transplanted during follow-up, 3 of whom were UNOS status 1.

**Figure 2:**
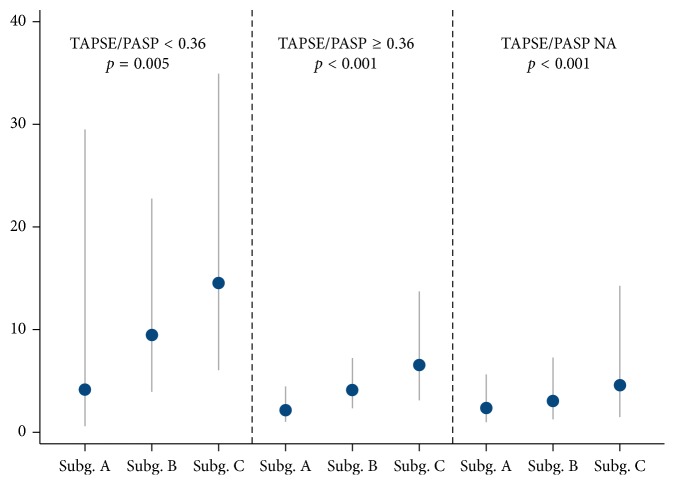
Predefined subgroup analysis: mortality rates per number of biomarkers and TAPSE/PASP categories (*p* for interaction = 0.24). Subgroup A (Subg. A): both biomarkers below the threshold; subgroup B (Subg. B): 1 biomarker above the threshold; subgroup C (Subg. C): 2 biomarkers above the threshold.

Except for EDT, all other candidate predictors were significantly associated with mortality on univariable analysis, with higher risks for older patients, women, and positive (above threshold) biomarkers; moreover, the risk increased linearly with the number of biomarkers (Table 3). We were not able to show an interaction of the number of biomarkers with the TAPSE/PASP ratio (*p*=0.24). In particular, at the predefined subgroup analysis, we observed a significant (and linear) increase in risk according to the number of biomarkers, with comparable slopes in all three TAPSE/PASP ratio categories (Figure 2).

In multivariable analysis including all candidate predictors, TAPSE/PASP ratio, number of biomarkers, and gender were independent predictors of death (Table 4, left panel): both the TAPSE/PASP ratio ≥0.36 and unavailable groups had a threefold decrease in risk of death with respect to the TAPSE/PASP ratio <0.36 group. The risk of death increased linearly by 1.6 for each additional positive biomarker and by almost 2 for women with respect to men. Age above 66 years was associated with a twofold increase in the risk of death, albeit (barely) not significantly. Model discrimination was fair, though not optimal (Harrell's *C* = 0.68). This model performed better (Harrell's *c* = 0.65) than one without biomarkers (Table 4, right panel).

## 4. Discussion

The main finding of our study is that, in HFrEF patients, the process of prognostic stratification based on echocardiographic parameters may be further refined using plasma levels of NPs and hsTnI.

Several previous studies have shown that echocardiographic and Doppler parameters play an important role in predicting cardiac events, including death, in patients with HF. First in 1999, in a population-based study, it was demonstrated that HFrEF patients who had received an echocardiographic evaluation had better survival and were more likely to be treated with ACE inhibitors than patients who were not assessed by echocardiography, even after adjusting for symptomatic status at presentation [23]. The restrictive pattern of left ventricular filling at pulsed-wave Doppler has been extensively validated as an indicator of poor prognosis [10–12]. More recently, the TAPSE/PASP ratio was shown to accurately stratify prognosis across the spectrum of HF, irrespective of ejection fraction [15]. The findings of our study confirm that the TAPSE/PASP ratio is a powerful independent predictive factor of mortality in HFrEF patients. Moreover, its acquisition is easy and fast, a non-negligible detail in daily clinical practice. However, the necessity of further refining the prognostic stratification after echocardiography is highlighted by the fact that, in our cohort of 431 patients, only 46 (11%) had a value of TAPSE/PASP ratio of <0.36, clearly pinpointing a cohort of more severely ill patients. In addition, PASP and therefore the TAPSE/PASP ratio could not be estimated in as many as 138 (32%) patients.

Though several biomarkers can be used to evaluate HF patients, most literature data refer to NPs and troponins. The use of NPs is valuable in differential diagnosis of patients that present with dyspnea to the emergency department [19]. Besides, NPs are well recognized as predictors of death or readmission after a first hospitalization for HF [24]. Data are also accumulating on potential usefulness of NPs in risk stratification of outpatients with chronic heart failure [25]. A significant limitation of NPs is represented by the lack of individualization of the therapeutic target. Indeed, the clinical stability of each patient may be expressed by different individual NPs values, the so-called dry NPs levels. For this reason, an attempt to reach a prespecified NPs level meaningful for all HF patients can be misleading and should not be recommended in current clinical practice. A consistent association has been demonstrated between hsTnI plasma levels and worse outcomes [18, 20, 26]. Although literature supports the use of biomarkers as risk indicators in the HF population, their use in combination with echocardiography has never been tested. This constitutes a gap in the evidence since echocardiography is the first and the most common imaging technique performed both for diagnostic purposes and in the follow-up of HF patients [14]. Since NPs and echocardiography can both reliably identify congestion, theoretically there might be an overlap between the information provided by biomarkers and echocardiography. At the same time, a synergism could also be hypothesized since hsTnI is a sensitive and specific marker of myocyte injury that cannot be detected at echocardiography.

For these reasons, in our population, we tested the potential prognostic role of both biomarkers. The results indicate that high plasma levels of NPs and of hsTnI are useful from a prognostic point of view in all HFrEF patients, regardless the initial risk identified by the echocardiographic examination. In other words, in both subgroups of TAPSE/PASP (high risk and low risk) and also in patients in whom this risk predictor was unavailable, knowledge of plasma levels of NPs and hsTnI could significantly improve the prognostic assessment of patients.

Therefore, this approach where echocardiography is used as the first tool to identify higher risk patients and is followed by the assessment of biomarkers can be applied to the most advanced HFrEF patients, for whom cardiac transplantation or mechanical assist device therapy should be considered. It can also be applied to a large part of the HF outpatient population in whom the TAPSE/PASP ratio is in the low risk range or is unavailable because PASP is not measurable in these patients.

### 4.1. Study Limitations

A number of study limitations have to be considered. First, the small cohort size and number of events do not allow for extensive multivariable models for predicting death. Specifically, given the limited number of events, the contribution of several comorbidities, including chronic obstructive lung disease, chronic kidney disease, and anemia, was not taken into consideration. However, we identified clinically important echocardiographic risk factors in our model, which were chosen *a priori*. We tested the variables of interest only against all-cause mortality so that we are unable to assess the risk for readmission for HF and/or heart transplantation. On the other hand, by choosing such a hard end point as all-cause mortality, we minimized the risk of error of adjudication of events. Another limitation concerns NPs levels: BNP was measured in 50% of patients in the study, and NT-proBNP was measured in the remaining patients. To overcome this limitation, we expressed NPs levels in dichotomized thresholds based on previous literature data for HF outpatients. Also, in our analysis, we did not include parameters of transverse RV shortening (like fractional area change) or of global RV myocardial longitudinal deformation (longitudinal strain) [27]; both of them seemed to us to be not part of the routine clinical practice in most laboratories of echocardiography. More specifically, global RV myocardial longitudinal deformation (longitudinal strain) was not feasible at the three participating centers when we conceived the present study. Finally, since the study was performed in tertiary referral centers for heart transplant, the patients enrolled in the study were on average much younger and had a lower prevalence of comorbidities than the most common heart failure patients in the real world.

In sum, The TAPSE/PASP ratio, easily measurable during a routine echocardiographic examination, emerged as a significant predictor of all-cause mortality in HFrEF patients and should be part of routine examination in this population. However, regardless of the degree of RV-arterial coupling impairment, the evaluation of plasma levels of both NPs and hsTnI can significantly contribute to identifying the patients with a worse prognosis. The results are thus in favor of using both echocardiographic data and plasma biomarkers to stratify the risk of all-cause mortality in stable HFrEF patients.

## Figures and Tables

**Table 1:** Clinical features of the study cohort.

Variable	All patients (*n*=431)
Age (years)	59 ± 12
Male gender, *n* (%)	357 (83)
Body mass index (kg/m^2^)	27 ± 4.5
Etiology, *n* (%)	
Ischemic	164 (38)
Nonischemic	267 (62)
Comorbidities, *n* (%)	
Hypertension	120 (28)
Diabetes	81 (19)
COPD	40 (9)
New York Heart Association class, *n* (%)	
I	89 (21)
II	265 (61)
III-IV	77 (18)
Systolic blood pressure (mmHg)	115 ± 18
Heart rate (beats/minutes)	68 ± 12
Rhythm, *n* (%)	
Sinus	329 (77)
Atrial fibrillation/flutter	35 (8)
Pacemaker	66 (15)
Glomerular filtration rate, MDRD (ml/min per 1.73 m^2^)	64 ± 17
Hgb (mg/dl)	13.9 ± 1.7
BUN (mg/dl)	46 ± 19
Medications, *n* (%)	
ACE-inhibitors/ARB	340 (77)
Beta-blockers	403 (93)
Aldosterone antagonist	246 (57)
Ivabradine	11 (3)
Digoxin	52 (12)
Furosemide (mg)	74 ± 100
ICD, *n* (%)	236 (55)
CRT, *n* (%)	98 (23)

COPD: chronic obstructive pulmonary disease; Hgb: hemoglobin; BUN: blood urea nitrogen; ICD: internal cardiac defibrillator; CRT: cardiac resynchronization therapy. Values are expressed as mean ± SD or *n* (%).

**Table 2:** Clinical features of the study cohort divided by TAPSE/PASP ratio <0.36, ≥0.36, or unavailable.

Variable	Patients (% of missingness)	TAPSE/PASP ratio <0.36 (*n*=44)	TAPSE/PASP ratio ≥0.36 (*n*=249)	TAPSE/PASP unavailable (*n*=138)	*p*
Age (years)	431 (0%)	60.2 ± 8.7	58.6 ± 12.4	58.7 ± 12	0.605
Age >66 years		11 (25)	67 (27)	37 (27)	0.987
Male gender, *n* (%)	431 (0%)	35 (80)	200 (80)	122 (88)	0.099
Body mass index (kg/m^2^)	430 (0.2%)	27.4 ± 4.2	26.5 ± 4	27.7 ± 5.3	0.150
Etiology, *n* (%)	431 (0%)				
Ischemic		24 (55)	89 (36)	51 (37)	0.061
Nonischemic		20 (45)	160 (64)	87 (63)	
Comorbidities, *n* (%)					
Hypertension	431 (0%)	13 (30)	55 (22)	52 (38)	0.005
Diabetes	430 (0.2%)	12 (27)	43 (17)	26 (19)	0.293
COPD	429 (0.5%)	4 (9)	18 (7)	18 (13)	0.156
NYHA class, *n* (%)	430 (0.2%)				
I		3 (7)	49 (20)	37 (27)	<0.001
II		27 (61)	149 (60)	89 (64)	
III-IV		14 (32)	50 (20)	12 (9)	
Systolic blood pressure (mmHg)	429 (0.5%)	108 ± 16	114 ± 16	121 ± 19	<0.001
Hgb (mg/dl)	389 (10%)	13.2 ± 2.1	13.7 ± 1.7	14.7 ± 1.3	<0.001
Creatinine (mg/dl)	382 (11%)	1.1 ± 0.2	1.2 ± 0.3	1.1 ± 0.2	0.346
NPs > threshold	431 (0%)	36 (82)	117 (47)	58 (42)	<0.001
hsTnI >0.027 pg/ml	431 (0%)	12 (27)	52 (20)	31 (22)	0.591
No. of biomarkers above threshold	387 (10%)				
0		8 (18)	113 (45)	65 (47)	0.004
1		24 (55)	103 (41)	57 (41)	
2		12 (27)	33 (13)	16 (12)	
Echocardiography					
LVEDVI (ml)	419 (3%)	117 ± 40	118 ± 42	125 ± 44	0.223
LVESVI (ml)	417 (3%)	107 ± 36	97 ± 38	101 ± 37	0.253
LA VOL	421 (2%)	123 ± 46	88 ± 43	85 ± 38	<0.001
LVEF (%)	431 (0%)	24 ± 6	27 ± 6	29 ± 5	<0.001
LVEF ≤35%		43 (98)	242 (97)	130 (94)	0.300
E/A	390 (9%)	2.12 ± 1.62	1.24 ± 0.77	1.09 ± 0.73	<0.001
EDT < 140 msec	422 (2%)	25 (58)	60 (24)	27 (20)	<0.001
MR yes	424 (2%)	27 (61)	97 (39)	46 (34)	0.008
PASP (mmHg)	298 (30%)^^^	55 ± 12	29 ± 9	26 ± 5	<0.001
PASP >40 mmHg	431 (0%)	39 (89)	31 (12)	0 (0)	<0.001
TAPSE ≤14	418 (3%)	22 (50)	20 (8)	14 (11)	<0.001

COPD: chronic obstructive pulmonary disease; NYHA: New York Heart Association; Hgb: hemoglobin; NPs: natriuretic peptides (brain natriuretic peptide, BNP, and N-terminal-proBNP, NT-proBNP); NPs thresholds are 125 pg/ml for BNP and 1016 pg/ml for NT-proBNP; hsTnI: high-sensitivity troponin I; LVEDVI: left ventricular end-diastolic volume index; LVESVI: left ventricular end-systolic volume index; LA VOL: left atrial volume; LVEF: left ventricular ejection fraction; E/A: ratio of mitral inflow E velocity to mitral inflow A velocity; EDT: mitral inflow E velocity deceleration time; MR: mitral regurgitation; PASP: pulmonary artery systolic pressure; TAPSE: tricuspid annular plane systolic excursion. ^^^Reasons of this rate of missingness are specified in Section 2. Values are expressed as mean ± SD or *n* (%).

**Table 3:** Candidate predictors and mortality (univariable Cox model).

Predictor	Number of deaths	Rate per 100 persons per year (95% CI)	HR (95% CI)	*p* value
TAPSE/PASP ratio				
<0.36	11	9.9 (5.5–17.8)	1	**<0.001**
≥0.36	26	3.6 (2.4–5.2)	0.34 (0.28–0.41)	<0.001
Unavailable	13	2.9 (1.7–5.0)	0.26 (0.12–0.56)	0.001
Number of biomarkers over threshold (NPs and hsTnI)				
0	13	2.3 (1.3–4.0)	1	**<0.001^^^**
1	22	4.3 (2.8–6.5)	1.86 (0.78–4.43)	0.162
2	15	7.3 (4.4–12.0)	3.01 (1.67–5.44)	<0.001
NPs				
≤Threshold	15	2.2 (1.3–3.6)	1	**0.006**
>Threshold	35	5.9 (4.2–8.2)	2.70 (1.32–5.49)	
hsTnI				
≤0.027 pg/ml	33	3.4 (2.4–4.8)	1	**<0.001**
>0.027 pg/ml	17	5.2 (3.2–8.4)	1.42 (1.18–1.70)	
EDT (msec)				
≥140	35	3.7 (2.7–5.1)	1	**0.593**
<140	13	4.1 (2.4–7.1)	1.08 (0.81–1.44)	
Age				
≤66	17	2.4 (1.5–3.9)	1	**0.032**
>66	33	5.8 (4.0–7.9)	2.37 (1.08–5.21)	
Gender				
M	38	3.5 (2.6–4.8)	1	**0.005**
F	12	5.9 (3.3–10.4)	1.75 (1.19–2.56)	

TAPSE: tricuspid annular plane systolic excursion; PASP: pulmonary artery systolic pressure; NPs: natriuretic peptides (brain natriuretic peptide (BNP), and N-terminal-proBNP (NT-proBNP)); NPs thresholds are 125 pg/ml for BNP and 1016 pg/ml for NT-proBNP; hsTnI: high sensitivity troponin I, threshold 0.027 pg/ml; EDT: mitral inflow E velocity deceleration time. ^^^Test for linearity of effect (*p*=0.809): there is a linear increase in risk of death with increasing number of positive biomarkers.

**Table 4:** Multivariable Cox model.

Predictor	Model with biomarkers	*p* value	Model without biomarkers	*p* value
	HR (95% CI)		HR (95% CI)	
TAPSE/PASP ratio				
<0.36	1	**<0.001**	1	**<0.001**
≥0.36	0.35 (0.29–0.43)	<0.001	0.29 (0.26–0.33)	<0.001
Unavailable	0.30 (0.12–0.72)	0.007	0.26 (0.12–0.55)	<0.001
Number of biomarkers over threshold (NPs and hsTnI)^^^			—	—
0	1	**<0.001**		
1	1.61 (0.79–3.25)	0.188		
2^*∗*^	2.60 (1.38–4.90)	0.003		
EDT (msec)				
≥140	1	**0.085**	1	**0.154**
<140	0.80 (0.61–1.03)		0.84 (0.66–1.07)	
Age				
≤66	1	**0.057**	1	**0.038**
>66	2.09 (0.98–4.48)		2.11 (1.04–4.25)	
Sex				
M	1	**0.002**	1	**0.001**
F	1.73 (1.22–2.45)		1.88 (1.32–2.68)	
*Modelpvalue*	<0.001	—	<0.001	—
*Harrell's c (95% CI)*	0.68 (0.60–0.77)		0.65 (0.54–0.76)	
*Akaike information criterion*	474		480	

TAPSE: tricuspid annular plane systolic excursion; PASP: pulmonary artery systolic pressure; NPs: natriuretic peptides; hsTnI: high-sensitivity troponin I; NPs: natriuretic peptides (brain natriuretic peptide (BNP) and N-Terminal-proBNP (NT-proBNP)); biomarker thresholds are >125 pg/ml for BNP, >1016 pg/ml for NT-proBNP, and >0.027 pg/ml for hsTnI; EDT: mitral inflow E velocity deceleration time. ^^^Test for linearity of effect (*p*=0.809): there is a linear increase in risk of death with increasing number of positive biomarker; ^*∗*^HR_[2 vs. 1]_ 1.62, 95% CI 1.31–2.00, *p* < 0.001.

## Data Availability

The data used to support the findings of this study are available from the corresponding author upon request.

## Acronyms

HFrEF: Heart failure with reduced ejection fraction
NPs: Natriuretic peptides
hsTnI: High-sensitivity troponin I
TAPSE: Tricuspidal annulus plane systolic excursion
PASP: Pulmonary artery systolic pressure
HF: Heart failure
RV: Right ventricle
HFpEF: Heart failure at preserved ejection fraction
LVEF: Left ventricular ejection fraction
CRT: Cardiac resynchronization therapy
EKG: Electrocardiogram
BNP: Brain natriuretic peptide
NT-proBNP: N-terminal pro-brain natriuretic peptide
EDTA: Ethylenediaminetetraacetic acid
MR: Mitral regurgitation
EDT: E-wave deceleration time
TR: Tricuspid regurgitation
RAP: Right atrial pressure
SD: Standard deviation
HR: Hazard ratio
UNOS: United Network for Organ Storage.
